# Molecular architecture of the autoinhibited kinesin-1 lambda particle

**DOI:** 10.1126/sciadv.abp9660

**Published:** 2022-09-16

**Authors:** Johannes F. Weijman, Sathish K. N. Yadav, Katherine J. Surridge, Jessica A. Cross, Ufuk Borucu, Judith Mantell, Derek N. Woolfson, Christiane Schaffitzel, Mark P. Dodding

**Affiliations:** ^1^ School of Biochemistry, University of Bristol, Biomedical Sciences Building, University Walk, Bristol BS8 1TD, UK.; ^2^ School of Chemistry, University of Bristol, Cantock’s Close, Bristol BS8 1TS, UK; ^3^ GW4 Facility for High-Resolution Electron Cryo-Microscopy, University of Bristol, Bristol, UK.; ^4^ Bristol BioDesign Institute, University of Bristol, Life Sciences Building, Tyndall Avenue, Bristol BS8 1TQ, UK.

## Abstract

Despite continuing progress in kinesin enzyme mechanochemistry and emerging understanding of the cargo recognition machinery, it is not known how these functions are coupled and controlled by the α-helical coiled coils encoded by a large component of kinesin protein sequences. Here, we combine computational structure prediction with single-particle negative-stain electron microscopy to reveal the coiled-coil architecture of heterotetrameric kinesin-1 in its compact state. An unusual flexion in the scaffold enables folding of the complex, bringing the kinesin heavy chain–light chain interface into close apposition with a tetrameric assembly formed from the region of the molecule previously assumed to be the folding hinge. This framework for autoinhibition is required to uncover how engagement of cargo and other regulatory factors drives kinesin-1 activation.

## Teaser

The microtubule motor kinesin-1 folds over at its elbow to form the compact autoinhibited lambda particle.

## INTRODUCTION

The autoinhibition of cytoskeletal motors is a fundamental regulatory mechanism that governs a vast array of cellular processes, ranging from intracellular transport to cell division and migration. These states are controlled by intramolecular or intracomplex interactions that serve to inhibit motor activity until it is required and prevent the wastage of adenosine 5′-triphosphate. There has been good progress in understanding the nature of the autoinhibited states of dynein and myosin family members in their higher-order functional complexes (*1*–*6*), but insight in the kinesin family has remained limited to isolated domains and certain critical interfaces (*7*–*12*).

**Fig. 1.**
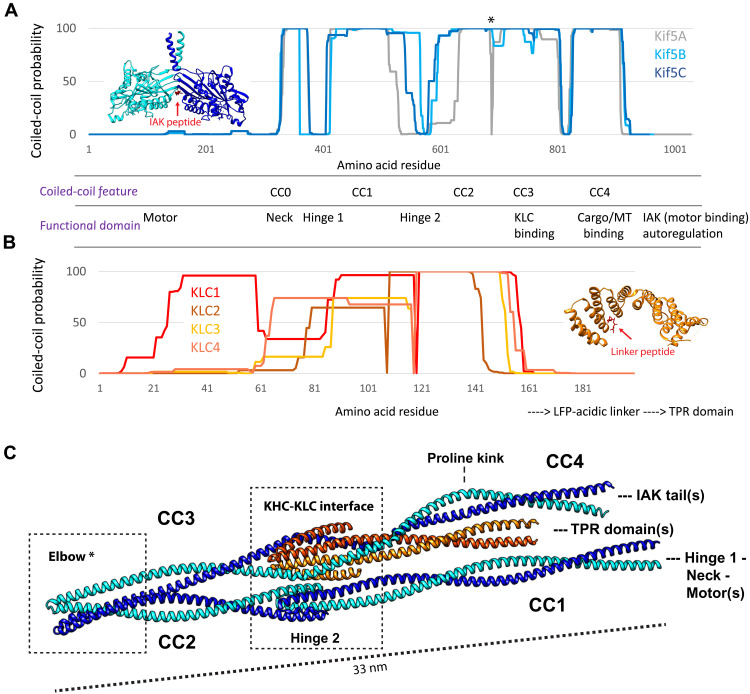
Computational prediction of a folded coiled-coil conformation for the kinesin-1 heterotetramer. (**A**) Coiled-coil probability plots generated using Marcoil for the three rat KHC paralogs KIF5A-C (NP_997688.1; NP_476550.1; NP_001101200.1). Inset shows the crystal structure of a motor domain dimer bound to a regulatory IAK peptide [*Drosophila* ortholog, Protein Data Bank (PDB): 2Y65] (*7*). Asterisk on the Marcoil plot indicates the position of the elbow feature. (**B**) Coiled-coil probability plots for the N-terminal regions of the four mouse KLC paralogs KLC1 to KLC4 (NP_032476.2; NP_001356289.1; NP_001272967.1; NP_001344059.1). Inset shows the crystal structure of TPR domain in the presence of its LFP (leucine-phenylalanine-proline)-acidic regulatory peptide (mouse KLC2, PDB: 5FJY) (*8*). (**C**) AlphaFold2-Multimer prediction of a 2:2 kinesin-1 heterotetramer composed of the CC1 to CC4 coiled-coil domains of rat KIF5C (NP_001101200.1, residues S410 to H917) and mouse KLC1 (NP_032476.2, residues T20 to S162). Positions of the globular domains shown in (A) and (B) are highlighted.

The ubiquitous and prototypic kinesin family member, kinesin-1, exists in a predominantly heterotetrameric form, comprising two motor-bearing heavy chains (KHCs) and two cargo-binding/regulatory light chains (KLCs) (*13*). As it carries out its diverse functions in the transport of vesicles, organelles, mRNAs, and multiprotein complexes, kinesin-1 transitions from a compact autoinhibited state to an extended active state. This is controlled by the binding of both cargo adaptors and microtubule-associated proteins (*8*, *14*–*23*). The compact state suppresses microtubule-dependent kinesin-1 adenosine triphosphatase (ATPase) activity because it allows a regulatory short-linear motif (SLiM) residing near the C terminus of the KHCs [known as the isoleucine-alanine-lysine (IAK) motif] (*7*, *24*–*30*) to bind the N-terminal motor domains (Fig. 1A). The KLCs also engage in a second regulatory SLiM-mediated self-interaction that occludes an important cargo adaptor binding site on their tetratricopeptide repeat (TPR) domains (Fig. 1B) (*8*, *31*, *32*).

Beyond these isolated interfaces, direct insights into the architecture of the autoinhibited state have been very limited since early pioneering electron microscopy (EM) studies outlined the domain organization and flexibility of the complex, including its sensitivity to ionic strength and pH (*33*–*37*). These conformational dynamics and resulting heterogeneity of isolated complexes are likely to be the main reason why progress has been limited. Hence, the organization of the series of KHC coiled-coil domains that extend throughout the complex remains a mystery. Consequently, it is unclear how the binding of the KLCs to these coiled coils helps to maintain the kinesin-1 complex in its inactive state (*15*, *23*, *38*). Here, we integrate computational structure prediction with EM to reveal the coiled-coil architecture of the autoinhibited compact conformer of the microtubule motor kinesin-1.

## RESULTS

### Computational prediction of a folded kinesin-1 coiled-coil assembly

We began by examining predictions of coiled-coil domains in the protein sequences of the three paralogous mammalian KHC (KIF5A, KIF5B, and KIF5C) and four KLC (KLC1 to KLC4) protein sequences using Marcoil coiled-coil prediction software (*39*, *40*). This highlighted a series of heptad repeats indicative of coiled-coil regions in KHC, CC0 to CC4 (Fig. 1A and fig. S1) (*41*): CC0 corresponds to the neck coil; CC1 to CC3 form the stalk, the latter component of which includes the KLC binding site; and CC4 is known to bind several cargoes/adaptors (*29*, *42*–*45*). CC1 and CC2 are separated by a drop in coiled-coil prediction probability (known as *Hinge 2*) (*26*, *28*, *46*, *47**)*, which has generally been thought to be the point where KHC folds in the autoinhibited state. The N-terminal sequence of KLC (which binds to KHC) also has heptad repeats and is predicted to form a segmented coiled coil. These are followed by an unstructured linker that leads on to the cargo-binding TPR domains (Fig. 1B and fig. S2). Consistent with their high degree of primary sequence homology, these coiled-coil predictions were similar across human, rat, and mouse sequences for the KHCs and KLCs (figs. S1 and S2).

To explore the structural organization of these coiled coils, we used AlphaFold2-Multimer (*48*, *49*) to predict a structure for a 2:2 heterotetramer consisting of CC1 to CC4 of KIF5C and the N-terminal domain of KLC1 isoform A (Fig. 1C and fig. S3). Consistent with the Marcoil prediction, the resulting model had CC1 to CC4 in KHC. Analysis of the model using SOCKET2 (*50*) identified the signature knobs-into-holes interactions of coiled-coil structures in these domains and provided a structure-based assignment of the heptad register (fig. S4). With the exceptions noted below, the coiled-coil domains are all canonical heptad-based parallel dimers, although CC4 had a noticeable proline kink (Fig. 1C and fig. S4). In addition, the location of the AlphaFold2-predicted light-chain binding site on CC3 is in good agreement with biochemical data, in the absence of a priori structural information (*51*).

However, we note three interesting and unexpected features in this model. First, the KHC-KLC interface predicts as a six-helix bundle rather than a tetrameric coiled coil previously assumed (*52*). The bundle is a dimer of antiparallel trimeric coiled coils formed from two N-terminal helices of the KLC and the C terminus of CC3 of KHC. Second, Hinge 2 falls in a parallel tetrameric coiled coil formed by the termini of CC1 and CC2, facilitated by a short helix in the loop that links them. Third, these two local assemblies are brought into apposition by a flexion in the KHC dimer between CC2 and CC3. We named this new feature the elbow, and it is marked in Fig. 1A with an asterisk. Comparison with the AlphaFold2 prediction of the KHC dimer without KLC (fig. S5) suggested that KLC may help to orient CC4, which is separated from CC3 by a triglycine (GGG) motif that kinks both assemblies but to different degrees (fig. S5). The length of the overall assemblies, at 33 nm long (N terminus to elbow), is in accordance with the first studies from Hisanaga *et al.* (*34*) that measured the distance of the flexion from the motor domains. Together, these data suggest the intriguing possibility that AlphaFold2 predicts the coiled-coil assembly of the autoinhibited ground state of the heterotetrameric complex.

### Isolation and visualization of the compact kinesin-1 conformer

**Fig. 2.**
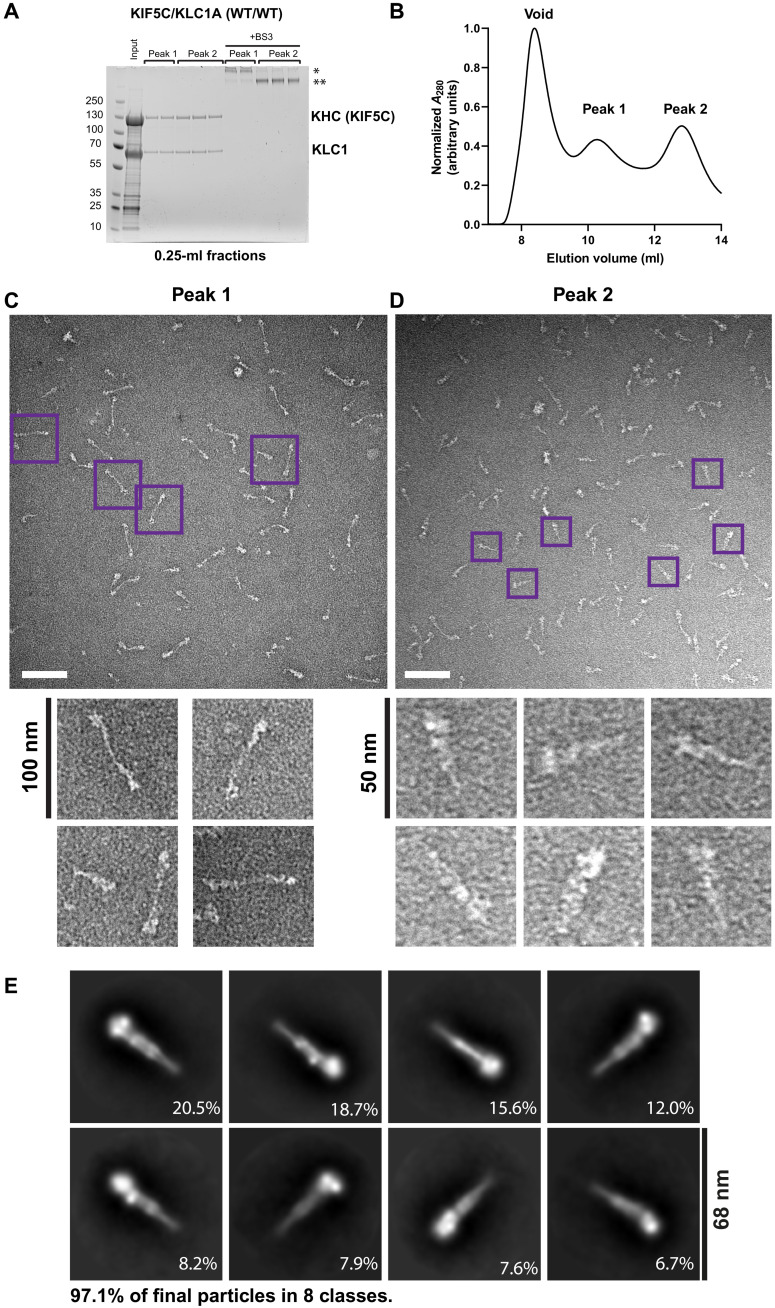
Negative-stain EM reveals the architecture of the compact the kinesin-1 conformer. (**A**) Coomassie-stained SDS-PAGE gels and (**B**) absorbance (*A*) measurements, showing SEC analysis of kinesin-1 (rat KIF5C/mouse KLC1A) complexes purified by nickel affinity chromatography. Complexes eluted in two distinct peaks after the void (peaks 1 and 2), and 0.25-ml fractions from across these peaks are shown. Lanes marked with +BS3 show the mobility of duplicate samples after cross-linking. Major and minor species referred to in the main text are marked with * and **. Data are representative of at least three independent experiments. (**C** and **D**) Representative T12 electron micrographs showing negative-stained, cross-linked sample from peak 1 (C) or peak 2 (D). Scale bars, 100 nm. Purple boxed particles are expanded below. (**E**) Reference free 2D class averages of compact peak 2 particles from the analysis of a cryo–negative-stain dataset.

To test the AlphaFold2 prediction, we isolated the full-length compact conformer of heterotetrameric kinesin-1 (KIF5C/KLC1 isoform A) from complexes expressed in *Escherichia coli* (Fig. 2, A and B). Compositionally indistinguishable KHC-KLC complexes eluted in size exclusion chromatography (SEC) in two peaks after the void, consistent with an equilibrium between two states (peaks 1 and 2; Fig. 2B) (*18*). To capture complexes in their post-elution state, the cross-linker bis(sulfosuccinimidyl)suberate (BS3) was added to the samples. For peak 1, this gave a major slowly migrating species on SDS–polyacrylamide gel electrophoresis (PAGE) (Fig. 2A, band marked by *) and a minor faster-migrating species (band marked by **). In contrast, for peak 2, the faster-migrating species was the more abundant, with only small amounts of the slower-migrating form. Together, these data suggested that proteins of peak 2 may be enriched for the compact conformer.

To characterize these fractions further, we turned to negative-stain EM. We found that the major species from peak 2 was a ≈40-nm-long V-shaped particle, with a wide end and a tapered end (82% of 279 particles counted were ≤45 nm in length) (Fig. 2, C and D). The remaining particles were longer and more heterogeneous. In contrast, preparations from peak 1 revealed predominantly long or bent thin particles of up to 80 nm in length, which is similar to the described open conformers (*18*, *33*–*35*, *37*), and relatively few of the ≈40-nm species (29% of 266 particles counted were ≤45 nm in length for peak 1 complexes).

**Fig. 3.**
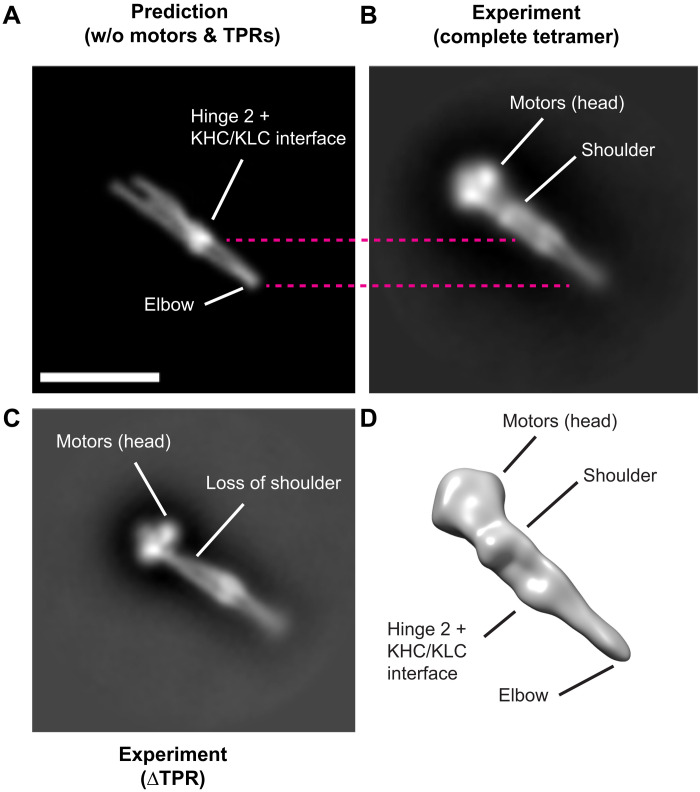
Comparison of computational and experimental data. Comparison of back-projection of the AlphaFold2 model (low-pass filtered to 30 Å) (**A**) with a 2D class average from experimental data for full-length (**B**) and ΔTPR complexes (**C**). All boxes are 68 nm. Scale bar, 25 nm. (**D**) 3D reconstruction from cryo–negative-stain EM of the full-length KIF5C/KLC1 tetramer from peak 2.

To study the compact species, a dataset of 3378 micrographs was collected from peak 2 grids and processed using RELION 3 (*53*). This yielded two-dimensional (2D) class averages consistent with a single particle (Figs. 2E and 3 and fig. S6). Notable features included a pair of globular densities, which—from their shape, size, and orientation—we attribute to the motor domains (*35*, *37*). Extending from the motor domains, at the head of the complex, there is a relatively thick rod along with additional globular densities, one close to the motor domains (marked “shoulder” on Fig. 3B) and one around halfway along the structure. The rod then tapers in a tight V shape to a point. To compare the AlphaFold2 model and 2D experimental data, low-resolution back-projection images (low-pass filtered to 30-Å resolution) were generated from the AlphaFold2 model (which does not include motor and TPR domains) (Fig. 3 and fig. S7). The predicted elbow feature could be identified unambiguously. The density toward the middle of the rod is concomitant with the predicted six-helix bundle at the KHC-KLC interface plus hinge 2. 3D reconstruction yielded a low-resolution envelope that supported our observations from the 2D classes and was consistent with the model (Fig. 3D).

### Deletion of the KLC TPR domains allows further definition of kinesin-1 structural features

To explore how the KLC TPR domains are positioned, we expressed and purified kinesin-1 complexes where the KLCs were truncated after the KHC binding heptad repeats (KLCΔTPR) (fig. S8). ΔTPR complexes eluted in two peaks in SEC, and cross-linked proteins from the two peaks showed comparable differences in electrophoretic mobility to those observed for the full-length proteins. Negative-stain EM analysis of peak 2 samples also revealed particles of similar size and morphology to those formed by full-length proteins, consistent with previous findings that the TPR domains are not essential to form the inhibited state (fig. S8) (*23*, *38*). Comparison of 2D classes to those of full-length proteins showed a clear and specific loss of the shoulder, enabling us to conclude that at least one of the KLC TPR domains forms the bulk of this feature (Fig. 3C).

### Computational analysis of the folding mechanism throughout the kinesin-1 family

We extended our computational analysis to include AlphaFold2 predictions of tetramers consisting of the coiled-coil domains of human KIF5A/KLC1, KIF5B/KLC1, and KIF5C/KLC1 (fig. S9). All predictions yielded the same global architecture: Complexes folded at the elbow with similar hinge 2 and KHC-KLC interface features, although with some local differences in structure. Analysis of the primary sequence of the elbow region in the KHC paralogs showed a high degree of conservation of the C terminus of CC2 and the N terminus of CC3, with divergence in the elbow loop (fig. S10). For KIF5A, this includes a proline residue at a *d* position in the Marcoil-predicted heptad repeat, explaining the uniquely low coiled-coil prediction probability for KIF5A at the */elbow position (Fig. 1A). We note the caveat that the evolutionary analysis performed by AlphaFold2 incorporates the highly homologous KIF5A, KIF5B, and KIF5C sequences in multiple sequence alignments and so may tend to converge on a similar fold. However, taking these models together with an independent analysis of the primary sequence (fig. S10), Marcoil coiled-coil predictions (figs. S1 and S2), and data showing that the three paralogs arose, in evolutionary terms, from relatively recent gene duplications in vertebrates (*54*), we expect that the elbow folding mechanism is conserved throughout the kinesin-1 family.

### Deletion of the elbow prevents formation of the compact kinesin-1 conformer

**Fig. 4.**
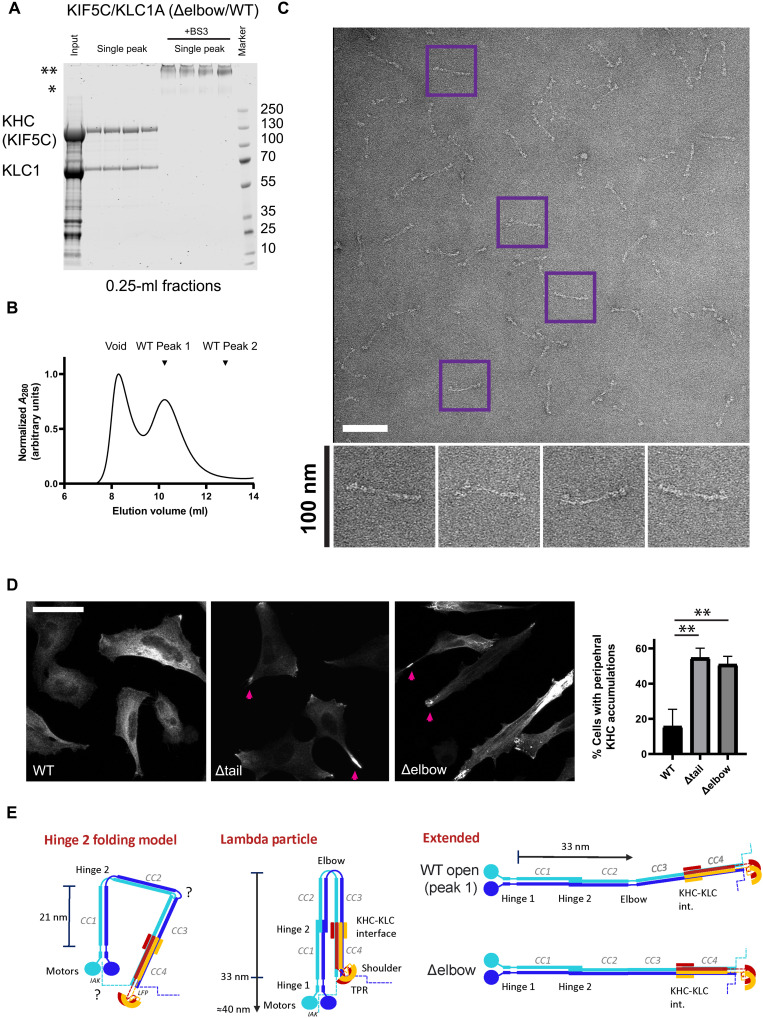
Disruption of the elbow prevents the formation of the compact kinesin-1 lambda particle. (**A**) Coomassie-stained SDS-PAGE gels and (**B**) absorbance measurements showing the results of SEC experiments with Δelbow kinesin-1 tetramers [KIF5C(Δelbow)/KLC1A(WT)]. BS3 lanes show the mobility of duplicate samples after cross-linking. Complexes eluted in a single peak after the void corresponding to the position of wild-type peak 1. Data are representative of at least three independent experiments. (**C**) Representative electron micrograph showing the negative-stained, cross-linked Δelbow/wt sample. Scale bar, 100 nm. Purple boxed particles are expanded below. (**D**) Immunofluorescence analysis of transfected HeLa cells expressing low levels of HA-KIF5C. Pink arrows show accumulations of Δelbow and Δtail proteins in the cell periphery. Scale bar, 25 μm. Graph shows quantification of the percentage of cells with prominent KIF5C accumulations in the cell periphery from three experiments (minimum of 25 cells per condition per experiment). **P < 0.01 using *t* test to compare the wild type to mutants. (**E**) Schematic illustrating previous Hinge 2 folding model and the new lambda particle model from the present study. Coiled coils are labeled as in Fig. 1 and are approximately to scale. The left schematic shows the challenge of rationalizing the Hinge 2 model with predicted lengths of coiled-coil sequences to form the compact conformer. Middle shows the lambda particle model derived from the present study. Blue dashed lines indicate the unstructured KHC C-terminal tails that contain the IAK regulatory motif. Right shows equivalent models for the extended conformers observed in this study.

As a further experimental test of the model, we sought to remove the elbow and join the flanking CC2 and CC3 regions of KIF5C as seamlessly as possible. The aim was to open the complex and so relieve autoinhibition. While the Marcoil and SOCKET2-assigned heptad repeats differ for this region (fig. S10), they concur in that the regular 3-4 alternating pattern of hydrophobic residues is broken. Closer inspection identified an 18-residue stretch rich in polar-charged residues, with a spacing between hydrophobics of 3-2-8, and spanning the turn predicted by AlphaFold2. Therefore, we removed this sequence to generate an Δelbow construct with a contiguous 3-4 hydrophobic spacing to join CC2 and CC3 (fig. S10). Consistent with this, kinesin-1 complexes with Δelbow eluted at the same position as wild-type peak 1 (Fig. 4, A and B), and negative-stain EM revealed almost exclusively extended particles of ≈80 nm long (only 2% of 229 particles counted were ≤45 nm in length) (Fig. 4C). Thus, the elbow is required to form the compact conformer. Last, consistent with motor activation, hemagglutinin (HA)–tagged KIF5C with Δelbow accumulated in the cell periphery, similar to complexes lacking the regulatory tail (Δtail) that cannot form the compact conformer (*30*), rather than displaying the predominantly diffuse cytosolic localization that is characteristic of wild-type complexes (Fig. 4D) (*55*).

## DISCUSSION

We suggest that the folded kinesin-1 conformer should be known as the kinesin-1 lambda particle (uppercase of the Greek letter, symbol Λ), after the autoinhibited dynein phi (φ) particle (*56*). The coiled-coil architecture revealed by the Λ-particle challenges the long-standing assumption that the complex folds on Hinge 2 (Fig. 4E) and provides the requisite molecular framework to discover how binding of cargo and regulatory factors controls the interconversion between this and the active state(s).

The Hinge 2 folding model was built on the finding that deletions of up to 105 amino acids in KHC spanning this feature resulted in increased motor activity measured by microtubule-dependent ATPase activity and single-molecule motility assays (*26*, *28*). However, we note that neither study reported the shift in sedimentation coefficient of the complex that would be expected from disruption of the folded conformer (*18*, *26*, *28*). More recently, Chiba *et al.* (*23*) concluded that deletion of Hinge 2 neither results in large-scale changes in the shape of the complex nor prevents light chain–mediated suppression of motor activity. Furthermore, it is challenging to reconcile the position of Hinge 2 and the predicted length of CC1 (approximately 21 nm) (Fig. 4E) with a flexion in the scaffold at >30 nm from the neck, as reported by Hisanaga *et al.* (*34*). Taking these findings in the context of our new model, we suggest that the increase in kinesin-1 activity from Hinge 2 deletion is adequately explained by a reduction in the combined length of the CC1-CC2 assembly of approximately 10 nm. This would alter the position of the motor domains with respect to the cargo-binding/regulatory tail in a manner that may not directly affect the ability of the complex to fold over but still disrupt tail-mediated suppression of motor activity (*26*, *28*).

The Λ-particle model also offers two plausible explanations for the role of the KLC coiled-coil domains in regulating motor activity (*23*, *38*) that are not mutually exclusive. First, KLC binding is predicted to orient CC4 toward the motor domains and so help to position the regulatory tail in proximity to the motor domains (Fig. 1C and fig. S5). Second, it suggests the possibility of cross-talk between Hinge 2 and the KHC-KLC interface; this may offer a mechanism by which autoinhibition can be disrupted. It is notable that the coiled coils immediately N-terminal (CC1) provide the binding site for the critical kinesin-1 cofactor MAP7/ensconsin that helps to recruit kinesin-1 to microtubules and promote activation (*19*–*21*, *23*, *57*–*60*). More broadly, numerous other regulators and cargoes have also been found to bind across the KHC and KLC coiled coils (*17*, *22*, *61*–*64*) that may alter or sense their conformational state.

Going forward, it will be important to determine when and where the compact conformer functions in a cellular context. As has been suggested previously, it may be that formation of the inhibited states may facilitate the transport or diffusion of the motors to sites of action (*65*). It may also be the case that inactive motors can associate directly with membranous cargo (*66*), in prime position for cargo adaptor–driven activation at the right place and time. Recent studies showing that amyotrophic lateral sclerosis (ALS)-linked mutations in KIF5A disrupt autoinhibition underscore the broad pathophysiological significance of these questions (*67*–*69*).

In summary, the computational prediction, isolation, and visualization of the Λ-particle coiled-coil assembly provide a long-sought coherent model for the formation of the compact conformer that explains the available data. It provides a platform on which to integrate new findings on the regulators and adaptors that control kinesin-1 activity in diverse cellular contexts and sets the stage for high-resolution structural studies of cargo-free and cargo-bound complexes, which will be required to take us toward an integrated understanding of kinesin-1–dependent intracellular transport and its dysregulation in disease.

## MATERIALS AND METHODS

### Plasmids and reagents

Rat KIF5C (corresponding to residues 2 to 955), was amplified by polymerase chain reaction (PCR) from an HA-tagged expression construct (*8*) and ligated into the His-3C cleavage pMW bacterial expression vector. Rat KIF5C ∆Tail (corresponding to residues 2 to 914) was amplified by PCR and inserted into HA-pCB6. Mouse KLC1 was amplified by PCR and ligated into pET28a using Nde I/Xho I sites. The N-terminal His-thrombin cleavage tag was removed by site-directed mutagenesis using the following primers: 5′-GAAGGAGATATAACCATGTATGACAACATGTCCACC-3′ (forward) and 5′-GTCATACATGGTTATATCTCCTTCTTAAAGTTAAACAAAATTAT-3′ (reverse). KLC∆TPR (KLC1A 1-162) was generated by addition of a stop codon using site-directed mutagenesis, using the following primers: 5′-GACATCTCCTAAACCCTCGGAGGACAAAGACTCT-3′ (forward) and 5′-GTCCTCCGAGGGTTTAGGAGATGTCGTCGTCGTA-3′ (reverse).

∆Elbow (KIF5C∆674–691) was generated by site-directed mutagenesis with the following primers: 5′-GCCCAACTGCAGGATGCTGAGG-3 (forward) and 5′-CTGCAGTTGGGCCCGGAGCTT-3′ (reverse). All plasmids were validated by DNA sequencing.

### Protein expression and purification

Heterotetrametric kinesin-1 was expressed in BL21(DE3) cells using a two-plasmid system. BL21(DE3) cells, transformed with both KIF5C and KLC1 expression plasmids, were used to inoculate 1 liter of LB cultures supplemented with both ampicillin and kanamycin. Cells were grown with shaking at 37°C until optical density reached 0.6 to 0.8 before the cultures were cooled to 18°C, and protein expression was induced with 0.3 μM isopropyl-β-d-thiogalactopyranoside. Following shaking incubation at 18°C overnight, cells were harvested by centrifugation at 6000*g* at 4°C for 15 min and resuspended (10 ml per 1 liter of original culture) in 20 mM Hepes (pH 7.4), 300 mM NaCl, and 40 mM imidazole before being stored at −20°C. Frozen pellets were thawed and diluted in 25 ml of buffer consisting of 40 mM Hepes (pH 7.4), 500 mM NaCl, 40 mM imidazole, 5% (v/v) glycerol, and 5 mM β-mercaptoethanol with a Roche complete protease inhibitor tablet added. Bacteria were lysed by sonication, 0.5 s on and 10 s off, at 70% amplitude for 7 min and 30 s in an ice bath. Lysate was clarified by centrifugation at 35,000*g* on a JA-20 rotor for 40 min at 4°C. Clarified lysate was filtered (0.45 μm) before being loaded onto a His-Trap (Sigma-Aldrich) column. Column was washed in base buffer and eluted with a gradient of base buffer of up to 500 mM imidazole. Eluted protein was concentrated by ultrafiltration in a 10,000-Da molecular weight cutoff filter (Cytiva), before snap-freezing in liquid nitrogen. Proteins were thawed and further purified by SEC using a Superose6 10/300 column (Cytiva), in 20 mM Hepes (pH 7.4), 150 mM NaCl, 1 mM MgCl_2_, 0.1 mM adenosine 5′-diphosphate, and 0.5 mM tris(2-carboxyethyl)phosphine (Sigma-Aldrich). For negative stain, freshly eluted proteins from size exclusion were cross-linked with 0.6 mM BS3 (Thermo Fisher Scientific) for 30 min at room temperature.

### Negative-stain EM

Freshly cross-linked proteins were diluted to 0.003 mg/ml in size exclusion buffer, and 5 μl was pipetted onto a freshly glow-discharged grid (300-mesh copper with formvar/carbon support, TAAB) and incubated at room temperature for 1 min. Grids were manually blotted and stained in 5 μl of 3% uranyl acetate (UA) for 1 min. The UA was blotted, washed in 3% UA, and a final 3% UA stain was applied for 30 s before blotting and allowing to air dry. Micrographs of grids were acquired on a FEI 120-kV BioTwin equipped with an FEI Ceta 4k x 4k charge-coupled device camera at ×49,000 magnification corresponding to a pixel size of 2.04 Å/pixel. Particle length was measured using ImageJ from several micrographs for each complex.

### Cryo–negative-stain EM dataset acquisition

Negative-stain grids were prepared as described above followed by cooling the grid liquid nitrogen immediately before loading into the microscope. Data were collected on a FEI Talos Arctica transmission electron microscope operated at 200 kV, equipped with a Gatan K2 Summit direct detector and Gatan Quantum GIF energy filter, operated in zero-loss mode with a slit width of 20 eV using the EPU software. Movies were acquired at a nominal magnification of ×130,000, giving a pixel size of 1.05 Å/pixel. A 100-μm objective aperture was used. For wild-type samples, a dataset of 3407 movies was collected in 55 fractions of 200 ms each. The total dose was 61.05 e^−^/Å^2^. For ∆TPR samples, 5268 movies were collected in 40 fractions of 150 ms each. The total dose was 59.34 e^−^/Å^2^. Movies were acquired with defocus values between −1.0 and −2.5 μm.

### Image processing

Image processing was performed in RELION 3.1.2 (*70*). The movies were motion corrected using MotionCorr2.1 (*71*). CTF estimation was performed using CTFFIND4 (*72*). For the wild-type dataset, motion-corrected micrographs were visually inspected manually, giving 3378 final micrographs, a representative example of which is shown in fig. S6. A total of 2008 particles were manually picked from a randomized 5% subset of micrographs. The manually picked particles were subjected to 2D classification, and the outputs were used to autopick the same 5% of micrographs, yielding 13,633 particles. The particles were subjected to several rounds of 2D and 3D classification. A resulting 3D model was used to autopick the entire dataset. This yielded 303,353 particles that were extracted and binned to 5.32 Å/pixel. These were subjected to further 2D and 3D classification, giving a final cleaned dataset of 22,440 particles (depicted by the 2D class averages present in Fig. 2). Particles were then reextracted without binning. All 22,440 particles were used to generate an ab initio initial model within RELION, which was then used as a reference for 3D classification with no symmetry imposed. A single 3D model was produced and subjected to autorefinement and postprocessing with a final reported resolution of 29.1 Å by gold-standard Fourier shell correlation (FSC) method (*73*). For ∆TPR data, 5268 movies were processed as for wild type. A total of 2084 particles were manually picked from a random 5% subset of motion-corrected micrographs and subjected to 2D classification. The outputs were used to autopick the same 5% of micrographs, yielding 58,234 particles that were subjected to multiple rounds of 2D classification. The resulting 2D classes were used to autopick the full dataset, giving 1,220,153 particles. Particles were extracted and binned to 5.32 Å/pixel, which, in turn, were subjected to further rounds of 2D classification, giving a final pool of 68,355 cleaned particles as depicted in fig. S8.

Back-projections of AlphaFold2 model were created by first projecting a volume using the MolMap command in UCSF Chimera. The volume was low-pass filtered to 30 Å, and back-projections were created with RELION’s “relion_project” tool.

### Cell culture and immunofluorescence

HeLa cells were grown and maintained in high-glucose Dulbecco’s modified Eagle’s medium (Sigma-Aldrich) supplemented with 10% fetal bovine serum (Sigma-Aldrich) and 1% penicillin/streptomycin (Gibco) at 37°C with 5% CO_2_. Transfections and immunofluorescence imaging were performed as previously described (*66*). Mouse anti-HA (HA-7) was from Sigma-Aldrich, and Alexa 568–conjugated anti-mouse was from Thermo Fisher Scientific.

### Marcoil coiled-coil analysis

Sequences with the accession numbers indicated in the figure legends were downloaded from the National Center for Biotechnology Information protein database (*74*) and processed using Marcoil coiled-coil prediction software that is part of the Max Plank Institute Bioinformatics Toolkit (*39*, *40*). Default parameters were used, and the output was processed for presentation in Microsoft Excel by downloading the coiled-coil probability list per residue and representing this as a graph.

### AlphaFold2 predictions

Predictions in Fig. 1C and fig. S5 were made using the Google Colab Alphafold Notebook with a Colab Pro+ subscription. The algorithm was asked to model a 2:2 heterotetramer composed of rat KIF5C (NP_001101200.1) residues S410 to H917 and mouse KLC1 (NP_032476.2) residues T20 to S162 or a homodimer of KIF5C. These boundaries were established by combining insight from Marcoil predictions shown in Fig. 1 and α-helical domain predictions of the monomeric proteins in the Alphafold-EBI structural database. Default parameters were used, and the amber relaxation step was enabled. The single-output model was downloaded in pdb format and prepared for presentation using PyMOL or UCSF Chimera (*75*). Model statistics are as presented by the software with annotations to highlight the relevant components. The additional models presented in fig. S8 were produced using the ColabFold Notebook (*76*). The sequences used are noted in the figure legend.

## Notes

View/request a protocol for this paper from *Bio-protocol*.
