# Supplementary material for: Molecular architecture of the autoinhibited kinesin-1 lambda particle
Source: Sci Adv. 2022 Sep 16;8(37):eabp9660. doi: 10.1126/sciadv.abp9660 (PMC9481135; doi:10.1126/sciadv.abp9660)
Supplement: Supplementary file 1 — Figs. S1 to S10 [file sciadv.abp9660_sm.pdf]

Science Advances
AAAS

Supplementary Materials for

Molecular architecture of the autoinhibited kinesin-1 lambda particle

Johannes F. Weijman *et al.*

Corresponding author: Mark P. Dodding, mark.dodding@bristol.ac.uk

Sci. Adv. **8**, eabp9660 (2022)
DOI: 10.1126/sciadv.abp9660

This PDF file includes:

Figs. S1 to S10

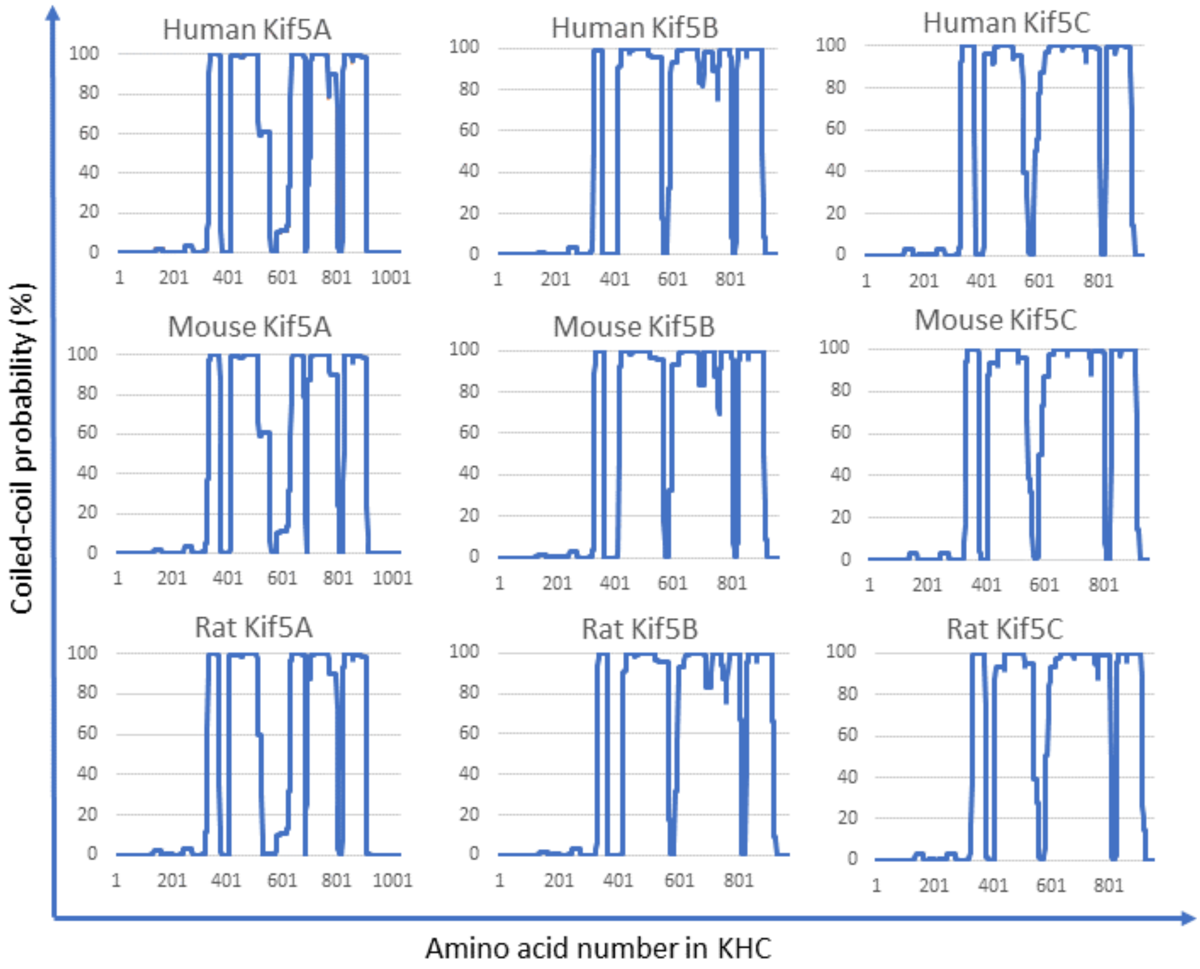

Fig. S1. Coiled-coil probability plots for the human, mouse and rat KHCs. Sequences were downloaded from the NCBI protein database and analysed using Marcoil. Accession numbers are: NP_004975.2-HsKIF5A; NP_004512.1-HsKIF5B; NP_004513.1-HsKIF5C; NP_001034089.1-MmKIF5A; NP_032474.2-MmKIF5B; NP_032475.2-MmKIF5C; NP_997688.1-RnKIF5A; NP_476550.1-RnKIF5B; NP_001101200.1-RnKIF5C. Hs – *Homo sapiens*; Mm – *Mus musculus*; Rn – *Rattus norvegicus*.

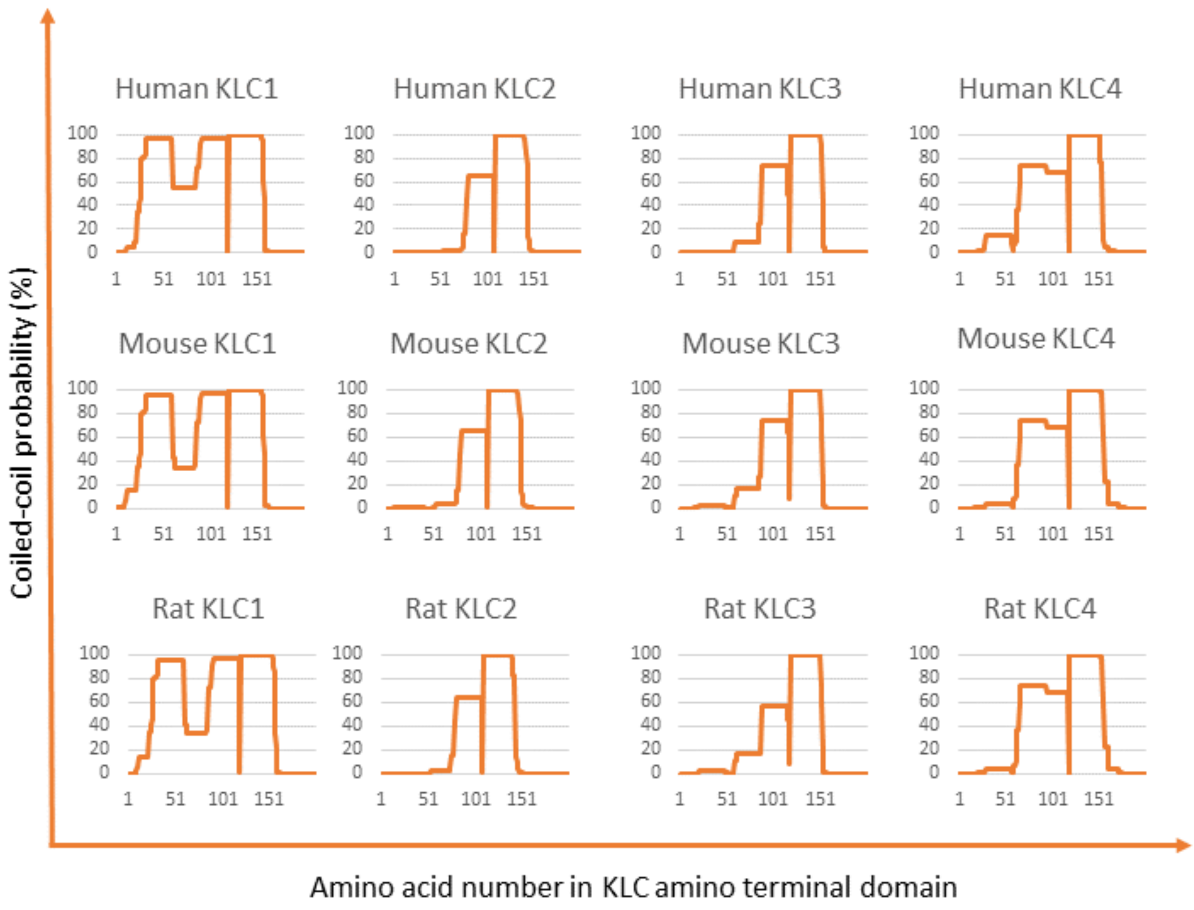

Fig. S2. Coiled-coil probability plots for the human, mouse and rat KLCs. Sequences were downloaded from the NCBI protein database and analysed using Marcoil. Accession numbers are: NP_001123579.1-HsKLC1; NP_001128247.1-HsKLC2; NP_803136.2-HsKLC3; NP_001275963.1-HsKLC4; NP_032476.2-MmKLC1; NP_001356289.1-MmKLC2; NP_001272967.1-MmKLC3; NP_001344059.1-MmKLC4; NP_001075441.1-RnKLC1; NP_001359013.1-RnKLC2; NP_612529.2-RnKLC3; XP_006244578.1-RnKLC4. Hs – *Homo sapiens*; Mm – *Mus musculus*; Rn – *Rattus norvegicus*.

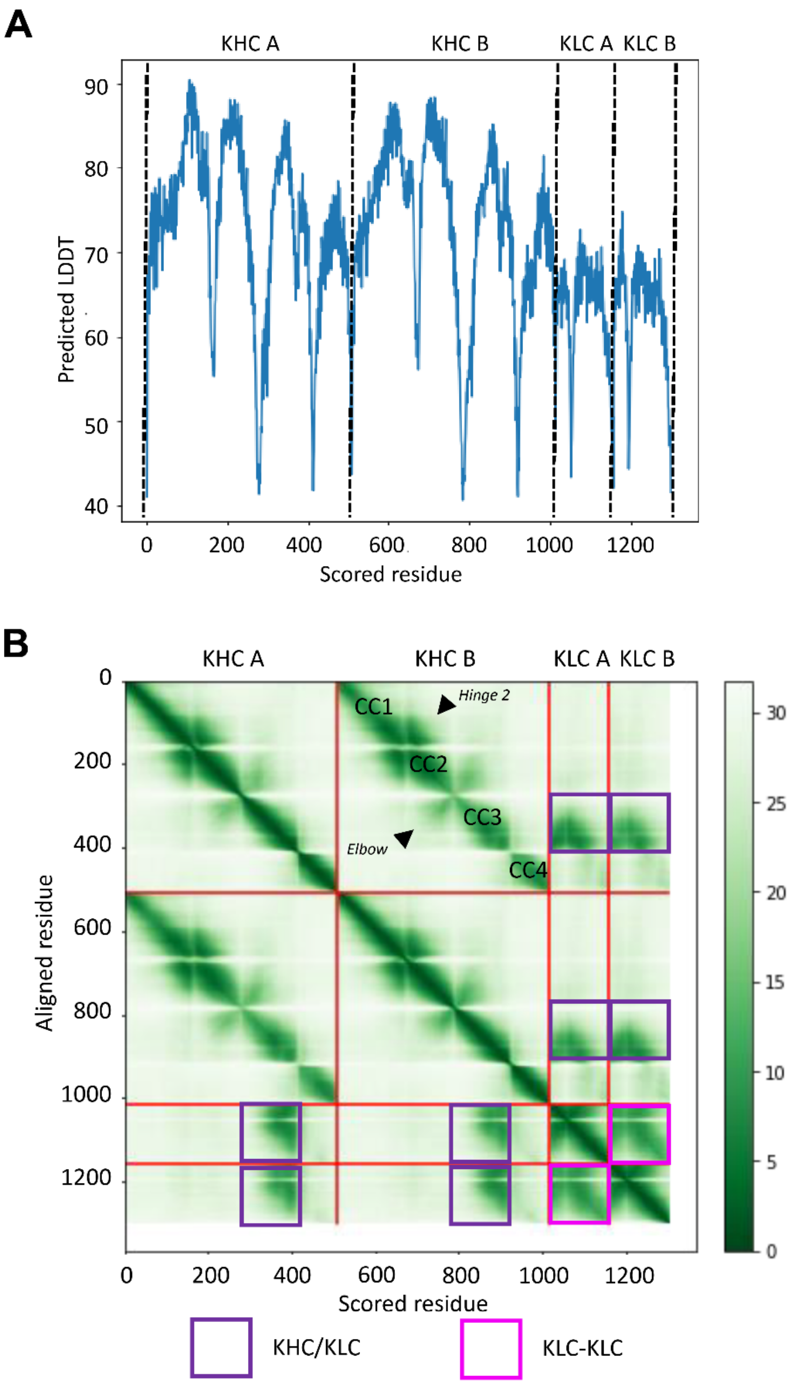

Fig. S3. AlphaFold2 model statistics for the KIF5C-KLC1 heterotetramer (A) Predicted local distance difference test (pLDDT) and (B) Predicted aligned error (PAE) plots for the AlphaFold2-Multimer KIF5C-KLC1 model presented in Figure 1C. Annotations are provided for features discussed in the main text.

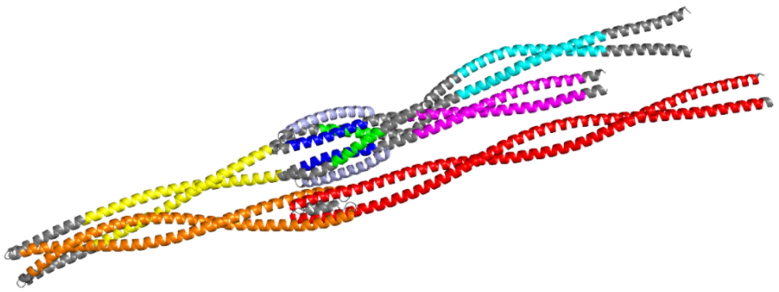

```
KIF5C 410-
SAEKEKYDEEITSLYRQLDDKDDEINQQSQLAEKLKQQMLDQDELLASTRRDYEKIQEELTRLQIENEAAKDEVK
   defgabcdefgabcdefgabcdefgabcdefgabcdefgabcdefgabcdefgabcdefgabcdefgabcde

EVLQALEELAVNYDQKSQEVEDKTRANEQLTDELAQKTTTLTTTQRELSQLQELSNHQKKRATEILNLLLKDLGE
fgabcdefgabcdefgabcdefgabcdefgabcdefgabcdefgabcdefgabcdefgabcdefgabcdefgabc
                                                               < Tetramer >
 <------  Hinge 2  ------>
IGGIIGTNDVKTLADVNGVIEEEFTMARLYISKMKSEVKSLVNRSKQLESAQTDSNRKMNASERELAACQLLISQ
defga              defgabcdefgabcdefgabcdefgabcdefgabcdefgabcdefgabcdefgabc
                      < Tetramer >
                                               <----- elbow ----->
HEAKIKSLTDYMQNMEQKRRQLEESQDSLSEELAKLRAQEKMHEVSFQDKEKEHLTRLQDAEEVKKALEQQMESH
defgabcdefgabcdefgabcdefgabcdefgabcdefgabcdefga

REAHQKQLSRLRDEIEEKQRIIDEIRDLNQKLQLEQERLSSDYNKLKIEDQEREVKLEKLLLLNDKREQAREDLK
   defgabcdefgabcdefgabcdefgabcdefgabcdefgabcdefgabcdefgabcdefga


GLEETVSRELQTLHNLRKLFVQDLTTRVKKSVELDSDDGGGSAAQKQKISFLENNLEQLTKVHKQLVRDNADLRC
     abcdefgabcdefgabcd                                abcdefgabcdefgabcdef
          AP Trimer

ELPKLEKRLRATAERVKALESALKEAKENAMRDRKRYQQEVDRIKEAVRAKNMARRAH-917
gabcdefgabcdefgabcdefgabcdefga

--------------------------------------------------------------------------
KLC1 20-
TQDEIISKTKQVIQGLEALKNEHNSILQSLLETLKCLKKDDESNLVEEKSNMIRKSLEMLELGLSEAQ
 abcdefgabcdefgabcdefgabcdefgabcde           abcdefgabcdefgabcdefgab
             AP Trimer                                  AP Trimer

VMMALSNHLNAVESEKQKLRAQVRRLCQENQWLRDELANTQQKLQKSEQSVAQLEEEKKHLEFMNQLK
cd              abcdefgabcdefgabcdefgabcdefgabcdefgabcdefgabcdefgabcd

KYDDDIS-162
```

Fig. S4. SOCKET2 structure-based assignment of the KIF5C and KLC1 heptad register. Structure shows the AlphaFold2 coiled-coil tetramer prediction in the same orientation as Figure 1C, here colored by coiled-coil domain predictions from SOCKET2. KIF5C and KLC1 sequences are shown below in the same color scheme with heptad register below. All are predicted parallel coiled coils with exception of the antiparallel (AP) and tetrameric structures highlighted in green below the heptad register. Sequences are rat KIF5C (NP_001101200.1) residues S410-H917 and mouse KLC1 (NP_032476.2) residues T20-S162.

.

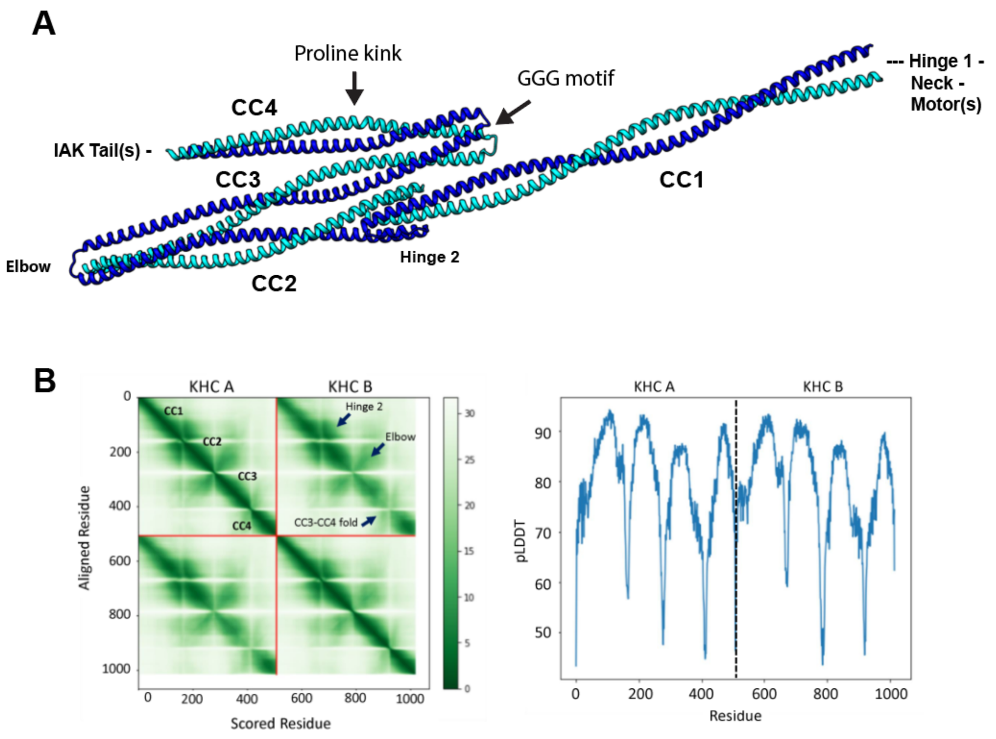

C

CC3 (KLC binding)

```
                                                                              Glycine motif

HsKif5A NP_004975.2         FLYERHEQSKQDLKGLEETVARELQTLHNLRKLFVQDVTTRVKKSAEMEPEDSGGIHSQK 827
HsKif5B NP_004512.1         VMQDRREQARQDLKGLEETVAKELQTLHNLRKLFVQDLATRVKKSAEIDSDDTGGSAAQK 829
HsKif5C NP_004513.1         LLNDKREQAREDLKGLEETVSRELQTLHNLRKLFVQDLTTRVKKSVELDNDDGGGSAAQK 831
RnKif5C NP_001101200.1      LLNDKREQAREDLKGLEETVSRELQTLHNLRKLFVQDLTTRVKKSVELDSDDGGGSAAQK 830
                            .: :::**:::*********::***************::******.*:: :* **  :**
```

CC4 (Cargo binding)

```
HsKif5A NP_004975.2         QKISFLENNLEQLTKVHKQLVRDNADLRCELPKLEKRLRATAERVKALEGALKEAKEGAM 887
HsKif5B NP_004512.1         QKISFLENNLEQLTKVHKQLVRDNADLRCELPKLEKRLRATAERVKALESALKEAKENAS 889
HsKif5C NP_004513.1         QKISFLENNLEQLTKVHKQLVRDNADLRCELPKLEKRLRATAERVKALESALKEAKENAM 891
RnKif5C NP_001101200.1      QKISFLENNLEQLTKVHKQLVRDNADLRCELPKLEKRLRATAERVKALESALKEAKENAM 890
                            ***************************************************.*******.*

                                                    Proline kink
```

Fig. S5. AlphaFold2 analysis of the KIF5C dimer (A) AlphaFold2-Multimer prediction of a rat KIF5C coiled-coil homodimer (NP_001101200.1) residues S410-H917. (B) Annotated PAE and pLDDT plots. (C) Clustal Omega multiple sequence alignment from KHC paralogues showing sequences of CC3 and CC4. The di/tri-glycine motif in the linker that separates CC3 and CC4 and the proline residue in CC4 are highlighted in yellow. Hs – *Homo sapiens*. Rn – *Rattus norvegicus*.

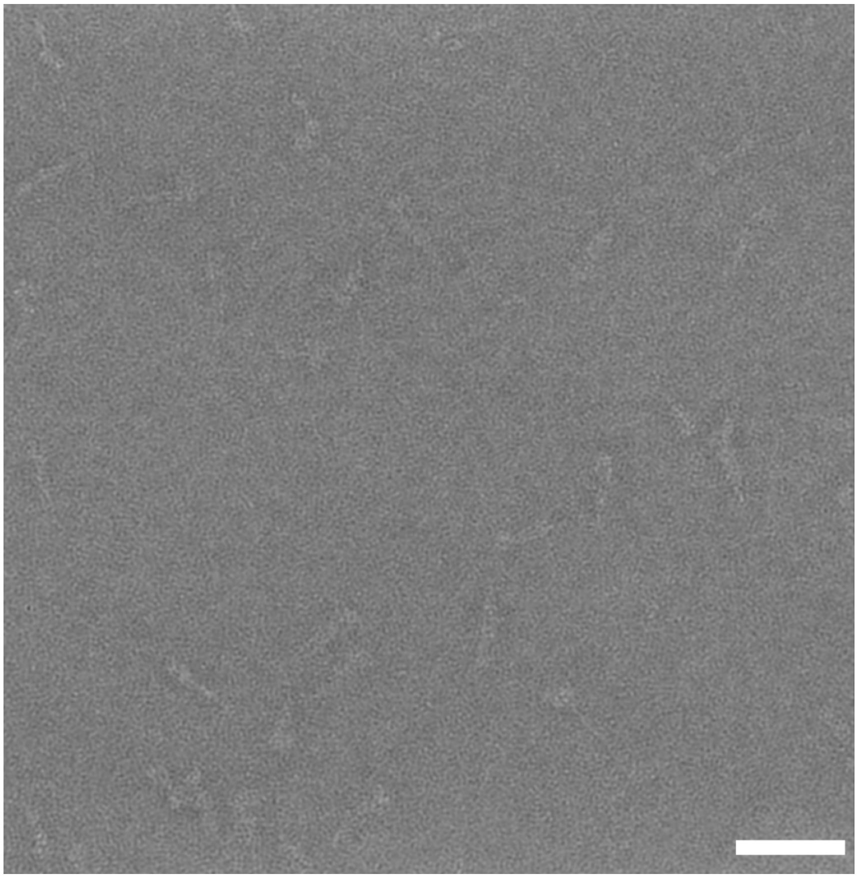

Fig. S6. Representative cryo-negative stain micrograph. Example of micrograph used for single particle classification of full length complexes. Scale bar is 50 nm.

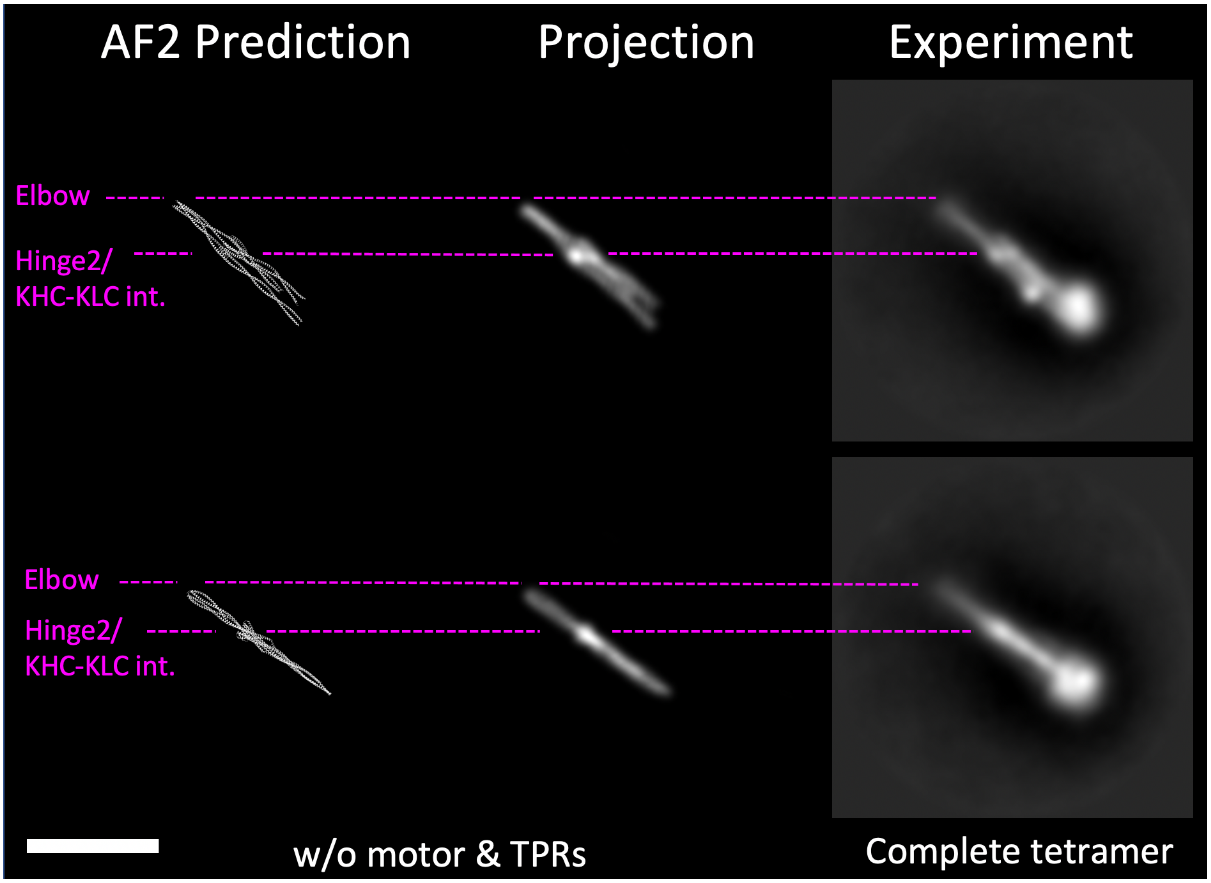

Fig. S7. Further comparison of computational and experimental data. Comparison of AlphaFold2 (AF2) atomic model, projection (low pass filtered to 30 Å) with 2D class from experimental data (full length complexes). Scale bar is 25 nm.

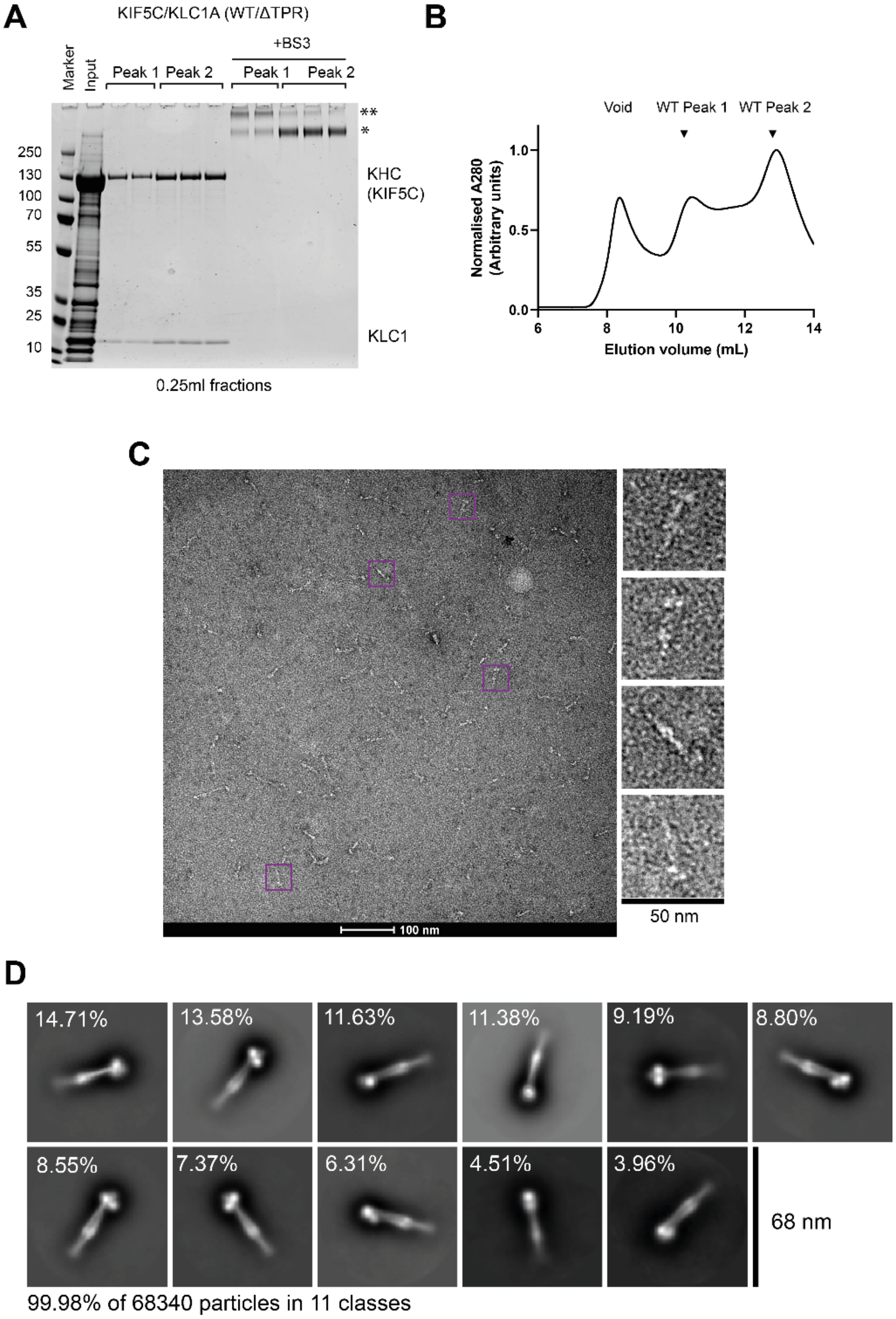

Fig. S8. Purification and electron microscopy analysis of complexes that lack KLC TPR domains. (A) Coomassie-stained SDS-Page gels and (B) absorbance measurements, showing SEC analysis of kinesin-1 (rat KIF5C / mouse KLC1A (ΔTPR)) complexes purified by nickel affinity chromatography. Complexes eluted in two distinct peaks after the void (Peak 1 and Peak 2) and 0.25ml fractions from across these peaks are shown. Lanes marked +BS3 show the mobility of duplicate samples after cross-linking. Major and minor species referred to in the main text are marked with * and **. (C) Representative T12 electron micrograph showing negative-stained, cross-linked sample from Peak 2. Purple boxed particles are expanded to the right. (D) Reference free 2D class averages of compact peak 2 particles from analysis of a cryo-negative stain dataset.

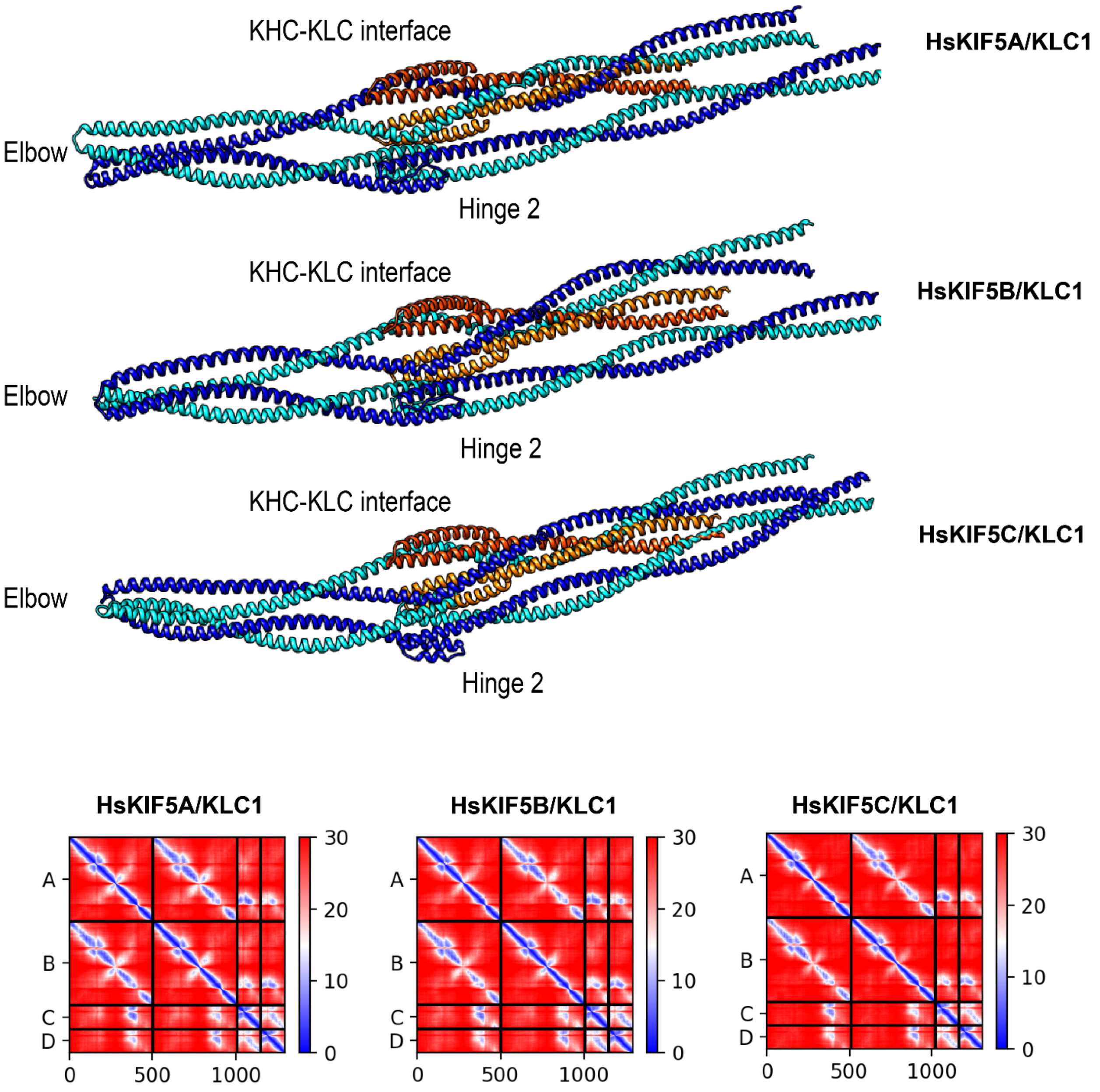

Fig. S9. AlphaFold2 models of human kinesin-1 complexes. Sequences were downloaded from the NCBI protein database. Accession numbers are: NP_004975.2 HsKIF5A (P411-H914); NP_004512.1 HsKIF5B (D415-H916); NP_004513.1 HsKIF5C (S410-H918); and NP_001123579.1 HsKLC1 (T20-S162). *Hs – Homo sapiens.* Models are shown above and PAE plots are provided below. Chains are labelled A and B are KHCs, chains labelled C and D are KLCs.

```
                       -------Δelbow----- -
651 -       QKRRQLEESQ DSLSEELAKL RAQEKMHEVS FQDKEKEHLT RLQDAEEVKK ALEQQMESHR EAHQKQLSRL RDEIEEKQRI IDEIRDLNQK - 740
MARCOIL     fgabcdefga bcdefgabcd efgabcdebc defgabcdef gabcdefgab cdefgabcde fgaefgabcd efgabcdefg abcdefgabc
SOCKET      fgabcdefga bcdefgabcd efgabcdefg a                                  defgabcd efgabcdefg abcdefgabc
AF2         HHHHHHHHHH HHHHHHHHHH HHHHHHHHHH HHHH-----H HHHHHHHHHH HHHHHHHHHH HHHHHHHHHH HHHHHHHHHH HHHHHHHHHH
PSIPRED4    HHHHHHHHHH HHHHHHHHHH HHHHHHHHHH HHHHHHHHHH HH-HHHHHHH HHHHHHHHHH HHHHHHHHHH HHHHHHHHHH HHHHHHHHHH
JPRED4      HHHHHHHHHH HHHHHHHHHH HHHHHHHHHH HHHHHHHHHH ----HHHHHH HHHHHHHH-H HHHHHHHHHH HHHHHHHHHH HHHHHHHHHH

            LKKRHLEESY DSLSDELAKL QAQETVHEVA LKDKEP---- DTQDADEVKK ALELQMESHR 707      KIF5A Hs NP_004975.2
            QKKRQLEESV DALSEELVQL RAQEKVHEM- ----EKEHLN KVQTANEVKQ AVEQQIQSHR 709      KIF5B Hs NP_004512.1
            QKRRQLEESQ DSLSEELAKL RAQEKMHEVS FQDKEKEHLT RLQDAEEMKK ALEQQMESHR 711      KIF5C Hs NP_004513.1
            QKRRQLEESQ DSLSEELAKL RAQEKMHEVS FQDKEKEHLT RLQDAEEVKK ALEQQMESHR 710      KIF5C Rn NP_001101200.1
             *:*:****  *:**:**.:* :***.:**:      *         * *:*:*: *:* *::***
```

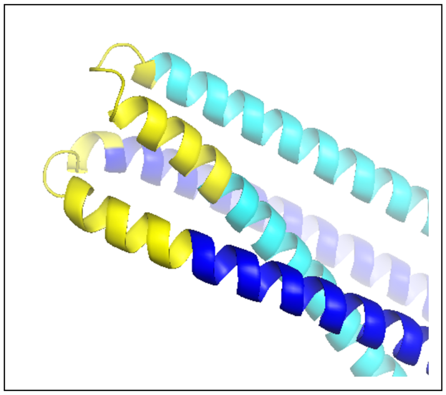

Fig. S10. Bioinformatic analysis of the kinesin-1 elbow. Alignment showing primary sequence of the KIF5C elbow region with Marcoil heptad prediction and SOCKET structure-based heptad assignment below in rainbow colors. The elbow deletion is highlighted in yellow on the sequence and also on the AlphaFold2 structure presented below (model as Fig. 1C). Secondary structure predictions from the AlphaFold2 model (AF2), Psipred4 and Jpred4 are also shown, along with a Clustal Omega multiple sequence alignment highlighting conservation and divergence of residues across this region in the KHC paralogues. A conserved proline residue within the elbow loop in KIF5A is highlighted in green. Hs – *Homo sapiens*. Rn – *Rattus norvegicus*.
